# Address of Usher Parsons, M. D., First Vice President of the National Medical Association, on Taking the Chair, at Its Recent Meeting at St. Louis

**Published:** 1854-07

**Authors:** 


### BUFFALO MEDICAL JOURNAL

AND

### MONTHLY REVIEW.

**VOL. 10.** JULY, 1854. **NO. .2**

### ORIGINAL COMMUNICATIONS.

ART. I.—*Address of Usher Parsons, M. D., First Vice President of the National Medical Association, on taking the chair, at its recent meeting at St. Louis.*

[We give below an authentic and full report of this excellent address. We have had the opportunity of submitting it to the personal inspection and correction of its distinguished author.—EDS.]

*Gentlemen of the American Association :*—It has been customary for the Presiding Officer of this Association, on retiring from the chair, to give a Valedictory Discourse. On the eve of my departure from Rhode Island, our venerable President notified me of his inability to attend and perform this part of his official duty, which deprives us of the rich entertainment anticipated from so distinguished a scholar and professor. The notice being entirely unexpected, I am unprepared to offer you anything worthy of your attention, and my inclination would therefore be to remain silent, but for an apprehension that this course might operate as a precedent to others on similar occasions. I will therefore present you as an apology for a discourse a few thoughts that have suggested themselves while on my way to this city.

In order to promote the honor, dignity and usefulness of our profession, objects for which the Association was instituted, its members must be gathered from all parts of our country, and united into one harmonious fraternity and must adopt such measures as will promote and perpetuate among ourselves an *esprit du corps*, a conformity of sentiment and feeling, and a combination and co-operation in action. This has already been accomplished in a good degree by holding our annual meetings in distant and remote cities of the Union. They must continue to be carried to new and ever varying spheres of action, until their beneficial influence is made available to the whole profession. As the metallurgist in separating a heterogeneous mass of particles passes over it a magnetic bar to attract the pure iron and steel with a force proportioned to its proximity, so must the meetings of this Association, in order to gather into one fold suitable materials of growth and strength, be carried from place to place over the whole mass of our whole population, attracting from the dross and impurities all that is of value and worthy of reception and incorporation into a homogeneous and efficient brotherhood.—These considerations influenced me in voting to accept the invitation to hold the present meeting in Missouri, notwithstanding the toil and fatigue of the journey, and its remoteness from the residence of a large proportion of the delegates. It is here, more than elsewhere, that the meetings of this Association are likely to prove beneficial by a rapid enlargement of our numbers.

Whoever glances at a map of the Mississippi valley, extending from the base of the Alleghany and Cumberland mountains to the margin of the Rocky Mountains ; from the highlands bordering on Lake Superior to the Gulf of Mexico, and contemplates the fertility of its soil, its adaptation for cereal productions, which are so necessary for human subsistence and increase, and who surveys the majestic Mississippi, navigable through this whole territory, with its numerous navigable tributaries pouring in their treasures on either side, and adds to this the vast mineral resources, lead, copper, iron and coal, which are far more conducive to healthful opulence than the golden regions of California—whoever, I say, candidly surveys all these elements of future growth, expansion and power, and moved onward by the agency of steam on land and water, and in labor-saving mechanical and manufacturing operations, can arrive at no other conclusion than that this vast territory, the largest and most favored one by nature of any under the whole canopy of Heaven, will, in time, be densely populated with scores of millions, and become the seat of empire of the western world ; and that it is destined to be the grand theatre of human progress in every department that is calculated to advance the dignity and promote the happiness of the human family.

And in no department of human affairs is progress here more sure than in medical knowledge. Our Atlantic States have inherited a reverence for European opinions, which, although commendable in our early medical history, is at the present day less favorable to American progress and discovery in medicine. We need to interrogate nature and experience more, and European opinions less. We need mental as well as political independence, a freer swing of thought and purpose that characterizes our brethren of the West, and which this Association is adapted to call into action.

There is much to encourage you in your recent discoveries and contributions, in the results of the vivisections of Saurians, the half of which, if confirmed by future experiments, will shed new light on Physiology ; and again in the discoveries made relating to the process of digestion by your late lamented Beaumont, of St. Louis, who, for the theories and speculations before prevailing, has substituted ocular demonstration of the *modus operandi* of that wonderful process, by submitting to it the various articles of human aliment, and determining the length of time required for converting each into healthful chyme; and again in the successful labors of Drake in traveling from state to state throughout the valley, collecting the history and character of its epidemics by personal inquiry and observation. Others of your venerated dead might be mentioned who have pursued a like independent course untrammeled by prevailing European authorities. Of their immediate successors who now stand at the head of their profession, it would ill-become me to speak seeing that some of them are present and unused to such freedom of remark. But to the junior members of the profession we would say, "unite, with us—follow the example of the distinguished pioneers I have named," and of Caldwell and Harrison who have gone to their reward; throw the result of your labors into the common stock of medical knowledge, accumulated by this Association, where, rest assured that they will be duly appreciated to the common benefit of the profession, and of mankind, and redound eventually to your everlasting honor and professional fame.

Gentlemen, eight years have elapsed since the preliminary meeting of the convention which recommended the formation of this National Association, and the results of its labors have equalled the expectations of the friends of reform and progress in our profession. The six published volumes of transactions have successively increased in value and interest, and are enduring monuments of the ardent zeal and patient industry of the numerous contributors, and there is every reason to hope that our future labors will continue to be crowned with equally increasing success.

Gentlemen, we are reminded by the history of the past year, of the frailty of human life. Death has removed many of our brethren of this Association. Among others, its first President, Professor Chapman of Philadelphia, a veteran teacher in our oldest Medical College—Professor Caldwell, another veteran of great distinction, as a lecturer and an author—Professor Howard, of Ohio, an eminent surgeon, editor of the Ohio Medical Journal, and who was Vice President of this Association at the time of his decease—Dr. George Shattuck, L. L. D., Boston, an extensive practitioner, and formerly President of the Massachusetts Medical Society; he was reputed the wealthiest physician in New England, and his numerous bequests to educational, humane and religious institutions, and private enterprises and charities, proclaim that his philanthropy was proportioned to his opulence.

We are reminded by the return of this anniversary of the terrible catastrophe that occurred at Norwalk. The Association had received from our, brethren of New York, a cordial welcome, and were honored with overflowing hospitality. After a delightful and profitable session, the Association adjourned, and many of the members were on their way in cars to their respective homes in joyous anticipation of rejoining their families, when in a moment, in the twinkling of an eye, seven of them were launched into eternity, leaving us the solemn admonition, that "in the midst of life we are in death." I should deem it a duty on this occasion to pay a tribute of respect due to their memory, by portraying their many virtues and excellences as men and as physicians, had I not ascertained that justice will be done them by an abler pen. Our brethren of New York, with characteristic magnanimity, which adds to their claim on our gratitude, immediately on the announcement of the disaster, summoned a meeting and passed resolutions expressive of their deep sorrow at the sad event; and they also appointed a committee to prepare an eulogy on the deceased to be offered at this annual meeting, and the distinguished ability of the chairman and members of that committee is a sufficient guarantee that justice will be done to the memory of these, our lamented brethren.

